# Genetic Variations Involved in Vitamin E Status

**DOI:** 10.3390/ijms17122094

**Published:** 2016-12-13

**Authors:** Patrick Borel, Charles Desmarchelier

**Affiliations:** NORT, Aix-Marseille Université, INRA, INSERM, 13005 Marseille, France; charles.desmarchelier@univ-amu.fr

**Keywords:** single nucleotide polymorphisms, genetic polymorphism, bioavailability, absorption, tocopherol, nutrigenetics, genome-wide association studies, candidate gene association studies, antioxidant, oxidative stress

## Abstract

Vitamin E (VE) is the generic term for four tocopherols and four tocotrienols that exhibit the biological activity of α-tocopherol. VE status, which is usually estimated by measuring fasting blood VE concentration, is affected by numerous factors, such as dietary VE intake, VE absorption efficiency, and VE catabolism. Several of these factors are in turn modulated by genetic variations in genes encoding proteins involved in these factors. To identify these genetic variations, two strategies have been used: genome-wide association studies and candidate gene association studies. Each of these strategies has its advantages and its drawbacks, nevertheless they have allowed us to identify a list of single nucleotide polymorphisms associated with fasting blood VE concentration and α-tocopherol bioavailability. However, much work remains to be done to identify, and to replicate in different populations, all the single nucleotide polymorphisms involved, to assess the possible involvement of other kind of genetic variations, e.g., copy number variants and epigenetic modifications, in order to establish a reliable list of genetic variations that will allow us to predict the VE status of an individual by knowing their genotype in these genetic variations. Yet, the potential usefulness of this area of research is exciting with regard to personalized nutrition and for future clinical trials dedicated to assessing the biological effects of the various isoforms of VE.

## 1. Vitamin E: The Major Lipid-Soluble Antioxidant in the Human Body

Vitamin E is the generic term for 8 natural isoforms that exhibit the biological activity of α-tocopherol: four tocopherols (α, β, γ, and δ) and four tocotrienols (α, β, γ, and δ). In this review, when we use the abbreviation VE instead of vitamin E, we refer to all these isoforms. α-tocopherol is the main vitamer found in the diet of Europeans, while γ-tocopherol is the main vitamer found in the diet of Americans, due to their higher consumption of soybean oil, which is a rich source of this vitamer. VE is present in numerous different foods but usually at low concentrations, except in vegetable oils and nuts. Therefore, it is difficult to meet the recommended dietary allowances for VE without eating these VE-rich foods, which are also rich in fat. The average α-tocopherol intake of Americans is still below the USA recommended dietary allowances (15 mg/day for people ≥14 years of age). In fact, about three-fourths of Americans (19–30 years of age) consume less than 10 mg/day [1]. In Europe, 8% of men and 15% of women fail to meet 67% of the European recommended dietary allowances for α-tocopherol [2]. As a consequence, a recent systematic review of the literature reporting vitamin E serum concentrations concluded that globally 13% of the subjects were below the functional deficiency threshold concentration of 12 μmol/L, mostly for newborns and children, and that only 21% of the subjects reached the threshold of 30 μmol/L, which is assumed to have beneficial effects on human health [3].

## 2. Vitamin E and Human Health

It is acknowledged that VE is the major lipid-soluble chain-breaking antioxidant found in the human body. Therefore, most studies dedicated to assess the biological effects of VE have focused on its ability to quench free radicals. They have confirmed that several VE isomers play a key role in the antioxidant defenses of our body. Nevertheless, some VE isomers have biological activities independent of their antioxidant properties [4]. For example, it has been shown that some VE isoforms can modify gene expression [5,6], inhibit cell proliferation [7], and modulate platelet aggregation [8] and enzyme activity by binding to the enzyme cofactor binding site [9].

Since oxidative stress has been involved in the etiology of cardiovascular diseases and cancers, the association between VE intake or VE status and the incidence of these diseases, as well as the potential benefit of VE supplementation on the incidence of these diseases, have been studied by several independent teams [10,11,12]. On the whole, although VE intake and VE status have been inversely associated with the incidence of these diseases, most randomized controlled trials have failed to show a benefit of α-tocopherol supplementation on the incidence of these diseases [13,14]. Several explanations have been offered to account for this discrepancy, such as opposing functions of VE isoforms (high plasma γ-tocopherol concentrations oppose the benefit of α-tocopherol [15,16]. It has also been suggested that the high interindividual variability of α-tocopherol bioavailability may have interfered with the protective effects of α-tocopherol supplementation [17,18,19]. This variability is at least partly due to genetic variations between individuals, and it is hence of paramount importance to identify these in order to take into account this confounding variable in future studies on the effect of VE intake or supplementation on various phenotypes.

## 3. Proteins Involved in Vitamin E Status

Fasting blood α-tocopherol concentration, measured in either plasma or serum, corrected or not by blood lipids, is regarded as the acknowledged marker of VE status. Two complementary approaches have been used to identify genetic variations that can modulate this status. The first one, in chronological order, is candidate gene association studies (CGAS’s). After an in silico search of candidate genes that are likely to affect α-tocopherol homeostasis because they are highly polymorphic [20], it has enabled the identification of the first genetic variants associated with the variability in α-tocopherol status [21,22]. Nevertheless, this approach has some drawbacks because many genetic variants with potential effect on VE status are left out of the analysis. Indeed, in CGAS’s, only a limited number of genetic variations, which are only present in the few genes selected by the researcher, usually through their known or putative involvement in the studied phenotype, are investigated. Besides requiring a thorough knowledge of the proteins and hence the genes that can affect the studied phenotype, selecting relevant genetic variants, i.e., genetic variants that significantly affect the activity or the expression of the candidate proteins, is also required. Although several tools are available to predict the physiological consequences of genetic variants, it is not yet possible to predict them with 100% accuracy. Thanks to progress in affordable high throughput genotyping techniques, genome-wide association studies (GWAS’s) have been increasing. One advantage of GWAS’s is that they do not make any assumptions on the genes or genetic variations that can affect the studied disease/phenotype—in this case, VE status. This allows researchers to identify associations that were not expected to be found. Nevertheless, GWAS’s also have their drawbacks. The main one is that the statistical stringency used in this approach, which is compulsory in order to avoid false positive associations, usually leads to false negative associations, i.e., to reject genetic variants that are actually associated with the studied disease/phenotype. This can be a problem in polygenic phenotypes/traits, such as VE status, which is affected by numerous proteins. Another drawback of GWAS’s is their cost. Since a very large number of genetic variations is investigated (typically >10^6^), the sample size required to limit false positive associations is high (typically >10,000 subjects). This is not necessarily an issue if the phenotype of interest is cheap to measure (e.g., one-point measurement such as fasting blood VE concentration), but this can become a limiting factor for more complex phenotypes such as the evaluation of VE bioavailability, which requires a postprandial experiment in a clinical environment with several measurement points. Finally, the studied populations need to be well characterized, obviously for the phenotype of interest but also for covariates that can influence this phenotype. For example, in the case of GWAS’s on the variability of VE status, not knowing the VE intake of the studied group greatly reduces the number of SNPs associated with the phenotype of interest since a significant part of the variability cannot be accounted for.

VE status is affected by numerous factors, e.g., dietary VE intake, VE absorption efficiency, and VE catabolism, but VE bioavailability has been shown to be a key determinant thereof [23]. Moreover, the knowledge of an individual’s VE absorption capacity is far more relevant than the knowledge of an individual’s fasting blood VE in order to provide personalized dietary recommendations. Since VE bioavailability is a phenotype that is relatively expensive to measure, the large sample size required to perform GWAS’s prevents the use of this approach to identify genetic variants associated with the variability in VE bioavailability. On the other hand, CGAS’s might be a good alternative since the limited number of genetic variants investigated does not require too large a sample size in order to obtain sufficient statistical power. It is thus important to provide a state of the art of the proteins that are known, assumed, or reasonably hypothesized to modulate, directly or indirectly, the bioavailability of VE. Indeed, future CGAS’s should focus on genetic variants in or near the genes that encode these proteins to increase their chance of finding significant associations. The last part of this chapter is therefore dedicated to listing these proteins by describing the fate of VE from the food matrix in which it has been ingested to its site of catabolism.

It is not the aim of this review to describe in detail the metabolism of VE in the human digestive tract, and more details on this topic can be found in recent reviews [24,25]. Gut VE metabolism might first involve gastric lipase, but there is no study dedicated to the assessment of whether this lipase is able to hydrolyze VE esters. Several studies have shown that α-tocopheryl esters are hydrolyzed to free α-tocopherol in the duodenum by carboxyl ester hydrolase [26]. The metabolism of VE in the gut lumen can also involve other digestive enzymes, such as pancreatic lipase, trypsin, and α-amylase, which can facilitate the release of VE from the food matrix and thus facilitate its micellization and thus its absorption. Nevertheless, there is no study dedicated to assessing whether they have a significant role in VE absorption.

After its release from the food matrix (usually dietary fat or a vegetable matrix), VE is incorporated into mixed micelles in the aqueous environment of the intestinal lumen. These lipid vehicles, which are mostly made of bile salts, phospholipids, cholesterol, and the products of triglyceride lipolysis, carry VE to the apical membrane of the enterocyte where is it absorbed. Although it was thought that VE uptake takes place by passive diffusion, several studies in cell cultures and in transgenic mice have demonstrated that proteins are implicated in this process [27]. To date, three proteins have been shown to be involved, directly or indirectly, in α-tocopherol uptake at the apical side of the enterocyte: scavenger receptor class B member 1 (SR-BI) [28], NPC1 like intracellular cholesterol transporter 1 (NPC1L1) [29,30], and CD36 molecule (CD36) [31]. After its uptake, VE has to reach the basolateral side of the enterocyte to be secreted in either chylomicrons or in intestinal HDL [32]. Surprisingly, there is no data yet on protein(s) involved in its intracellular transport. Yet, α-tocopherol is able to bind to bovine serum albumin [33]. Candidates could be Niemann–Pick type C1/C2 (NPC1/2) and sec14p-like proteins (encoded by transporter 1, ATP binding cassette subfamily B member (*TAP*1, 2, and 3)), which transport α-tocopherol in other cells. It is assumed that most, if not all, VE is incorporated into chylomicrons, but it seems that a fraction of α-tocopherol is secreted in intestinal HDL via a basolateral membrane protein, ATP binding cassette subfamily A member 1 (ABCA1) [34]. The incorporation of VE within chylomicrons likely depends on several proteins, including microsomal triglyceride transfer protein (MTP) [32], which is required for the incorporation of triglycerides within the nascent chylomicrons. Chylomicron VE and intestinal-HDL VE are then transported to the liver and to other tissues. Apolipoproteins that belong to these lipoparticles, e.g., apoB48 and apoAI, as well as proteins that are involved in the blood metabolism of these lipoparticles, e.g., lipoprotein lipase, cholesteryl ester transfer protein (CETP), phospholipid transfer protein (PLTP), and apoB receptor, are also involved indirectly in VE metabolism [21].

The fate of VE that is incorporated into intestinal HDL is not known, but it is assumed that most of this VE is transported to the liver where it is taken up via SR-BI. Concerning chylomicron VE, it is assumed that, thanks to the action of lipoprotein lipase which can release some of the VE entrapped into chylomicron triglycerides, a fraction is taken up by extrahepatic tissues [35]. The fraction of VE that remains in the chylomicron remnants is taken up by the liver via endocytic receptors, such as LDL receptor and heparan sulfate proteoglycans. The liver is a hub for VE metabolism. Indeed, it can store VE, secrete it in the bile or in very-low-density lipoproteins (VLDL), or catabolize it. The regulation of these different pathways is not known, but we hypothesize that this is at least partly dependent on the VE status. Only one protein involved in these regulations has been identified: α-tocopherol transfer protein (α-TTP), which is required for the incorporation of α-tocopherol into VLDL and which is mostly responsible for the different fate of VE isomers in the body. Indeed α-TTP, which was first isolated from bovine liver [36], preferentially bind RRR-α-tocopherol among all other VE isomers [37]. Loss-of-function mutations in the gene that encodes α-TTP strongly impair the transfer of α-tocopherol into VLDL, resulting in a disease called “ataxia with isolated α-tocopherol deficiency”, demonstrating the key role of this protein in the control of blood α-tocopherol concentration. It is not known whether this protein allows the liver to pilot the VE isoforms to the other above-mentioned pathways, or whether it is involved in the protection from β-oxidation [38], but no other protein putatively involved in these pathways has been identified. Concerning VE catabolism, one enzyme has been identified: ω-hydroxylase cytochrome P450-4F2 (CYP4F2); although another cytochrome, CYP3A4, has been suspected to play a role, CYP4F2 is apparently the only one involved in α-tocopherol catabolism in humans [39].

**Figure 1:**
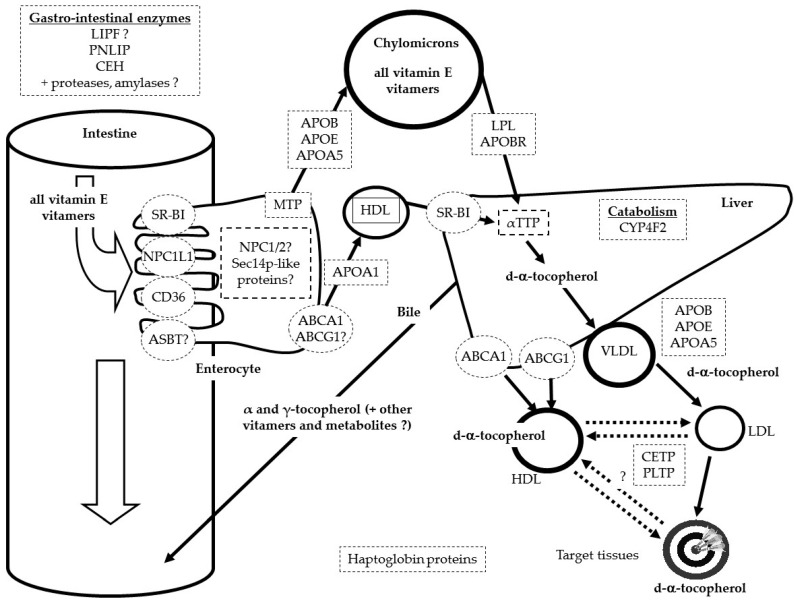
Summary of the proteins involved in the variability of vitamin E (VE) status. LIPF (gastric lipase), CEH (carboxyl ester hydrolase), ASBT (human bile acid transporter), LPL (lipoprotein lipase), APOBR (apolipoprotein B receptor), ABCB1 (ATP binding cassette subfamily B member 1).

The fate of VE in lipoproteins is not accurately known. It is assumed that most VLDL-VE stays in these lipoparticles during their hydrolysis by lipoprotein lipase. Thus, VE present in LDL is assumed to come mainly from VLDL. Nevertheless, it has been shown that a fraction of VLDL- and LDL-VE can transfer between these lipoproteins and HDL, and vice versa. This mass transfer is facilitated by PLTP [40,41] and likely also by CETP [42]. It is assumed that a fraction is degraded by oxidation when VE reacts with free radicals and that the remaining fraction is either taken up by the liver or by other tissues during lipoprotein uptake. The fate of the various isomers of VE in the various tissues of the body is barely known. It is only assumed that a significant fraction is stored in the lipid droplets present in adipose tissue [43,44,45] and that another fraction is incorporated in cell membranes where it can protect lipids from oxidation [46,47] or modulate phospholipid externalization [48]. It is finally assumed that tissue VE is either degraded by oxidation or is naturally catabolized. Yet, a fraction can be transported back to the liver via HDL, mimicking the reversal transport of cholesterol [49]. Because it is obvious that oxidative stress plays a key role in the blood concentration of this antioxidant, the last candidate proteins/genes that could modulate blood VE concentrations are those involved in the production/neutralization of reactive oxygen species. Indeed, the blood/tissue concentration of these species can modulate the blood/tissue concentration of VE. This explains why genetic variants in the haptoglobin proteins have been associated with the variability in α-tocopherol status [18]. Figure 1 shows the proteins assumed to participate in the transport of VE from the gastro-intestinal lumen to its target tissues.

Proteins displayed are those encoded by the main candidate genes involved in VE status or by genes for which single nucleotide polymorphisms have been associated with the variability of VE status. Proteins followed by a question mark have yet to have their implication confirmed. Metabolites secreted in the urines, e.g., 3′-carboxychromanol metabolites (α- and γ-carboxyethyl hydroxychroman), are not shown.

## 4. Genetic Variations Associated with the Variability in Vitamin E Status

As stated previously, both GWAS’s and CGAS’s have been applied to identify SNPs associated with fasting blood VE concentrations. Three GWAS’s have shown that a SNP in *CYP4F2*, a SNP in *SCARB1*, and a SNP near *APOA1/C3/A4/A5* are associated with α-tocopherol status [50,51,52]. As mentioned above, *CYP4F2* encodes for cytochrome P450 4F2, which catabolizes VE. *SCARB1* encodes for SR-BI, which is a plasma membrane receptor for HDL and which has been involved in α-tocopherol uptake by several tissues [28,53]. It is therefore not surprising that genetic variants in these genes can modify blood VE concentration. Concerning the association of a SNP within the cluster *APOA1/C3/A4/A5*, which is in linkage disequilibrium with *ZNF259* and *BUD13* that have no known role in α-tocopherol metabolism, it is likely due to a genetic variant in *APOA5*, as suggested by Ferrucci et al. [52]. CGAS’s have confirmed the associations found in the GWAS’s [21,22,54,55,56]. Furthermore, they have showed that genetic variants in other genes, i.e., *CD36* [57], *CETP* [21] and *APOE* [22], are also likely involved in VE status. Although, as explained above, the fact that these associations were not observed in GWAS’s suggests that their effect on VE status is moderate.

The following table (Table 1) summarizes all the genetic variants that have been associated with the variability in vitamin E status.

## 5. Genetic Variations Associated with the Variability in Vitamin E Bioavailability

Fasting blood α-tocopherol concentration, which is usually used to evaluate VE status, is affected by numerous factors, such as dietary intake of VE, dietary intake of pro-oxidant compounds, and α-tocopherol metabolism within the body. Although the relative role of each of these factors is unknown, it is assumed that the effect of the absorption efficiency of VE is very important with regard to long-term VE status. This assumption is supported by the fact that a significant relationship between α-tocopherol status and α-tocopherol bioavailability has been observed [23]. More precisely, in a recent clinical trial from our group where an α-tocopherol-rich meal, which provided VE as 67 mg (100 IU) d-α-tocopheryl acetate, was given to a group of healthy subjects, the coefficient of variation of the α-tocopherol response to the test meal, i.e., the postprandial chylomicron α-tocopherol response, an acknowledged estimate of α-tocopherol bioavailability, was 81% [23]. In this study, the interindividual variability in α-tocopherol bioavailability was associated with a combination of 28 SNPs in or near 11 candidate genes. Seven of these genes were involved in the postprandial chylomicron triacylglycerol response in the same group of subjects [59], which was not surprising, as most newly absorbed VE is carried from the intestine to the liver via chylomicrons. Four of these genes were specifically associated with α-tocopherol response, suggesting that they play a specific role in α-tocopherol bioavailability. These genes were *SLC10A2* (solute carrier family 10 (sodium/bile acid cotransporter), member 2), *PNLIP* (pancreatic lipase), *SREBF2* (sterol regulatory element binding transcription factor 2), and *ABCG1* (ATP-binding cassette, sub-family G (WHITE), member 1). We hypothesize that genetic variations in *SLC10A2* are associated with α-tocopherol bioavailability because either the protein encoded by this gene is involved in α-tocopherol transport across membrane of the enterocyte, or because this protein is involved in bile salt absorption, which has an indirect effect on the absorption of VE incorporated in bile-salt containing micelles. Genetic variations in *PNLIP* likely have an indirect effect on VE micellization because dietary VE is usually embedded in dietary fat and triglyceride hydrolysis by pancreatic lipase is assumed to facilitate the release and the transfer of VE from the oil droplets of dietary lipid emulsions to mixed micelles. *SREBF2* encodes for a transcription factor that controls the expression of *NPC1L1*, among other genes. Since NPC1L1 is involved in α-tocopherol uptake by the intestinal cell [29,30], it is likely that genetic variants in *SREBF2* can indirectly affect α-tocopherol absorption. *ABCG1* encodes for a membrane transporter of various molecules across cellular membranes. Thus, the association observed between genetic variants in this gene and α-tocopherol bioavailability suggests that this protein is able to transport α-tocopherol as well. This was confirmed recently by a study in ABCG1-deficient mice [60]. Although there has only been one study dedicated to identifying the genetic variants involved in α-tocopherol bioavailability, and although we acknowledge that the results obtained should be confirmed in other groups of subjects, we confidently conclude that it is likely that several SNPs in different genes have an effect of VE bioavailability. Moreover, although the effect of each SNP can be relatively low (e.g., 1%–2% of the variability in α-tocopherol bioavailability explained), their additive effect can have a significant effect on bioavailability.

## 6. Other Genetic Variations Potentially Involved in Vitamin E Status

As stated in this review on the genetic variations involved in VE status, much work remains to be done. Indeed, GWAS’s only allow us to identify SNPs associated with the VE status in large populations where covariates (e.g., VE intake and smoking status) are well measured while CGAS’s can miss out SNPs in genes not thought to affect this status. It can also be concluded that, since VE status is modulated by numerous genes, it is necessary to simultaneously know the effect of several genotypes in order to try to predict VE status with genotyping. It should also be reminded that SNPs are not the only genetic variations that occur in DNA. Indeed, there are also copy number variants, insertion/deletion of certain base pairs, and epigenetic modifications, e.g., DNA methylation. A genetic score that would aim to predict VE status should therefore take into account all the genetic variations that can have a significant impact on VE status. Furthermore, association studies have to be performed in different populations to be sure that the associations are not specific to certain ethnic groups.

In summary, there is now enough evidence to state that VE status is partly modulated by SNPs in several genes. Although much work remains to be done to obtain a combination of genetic variations (SNPs but also other kinds of genetic variations) that will allow us to confidently predict the VE status of an individual by knowing his genotype at these variations, the potential usefulness of this area of research is exciting with regard to personalized nutrition and for future clinical trials dedicated to assess the biological effects of VE. Nevertheless, it should be reminded that genetics only represents one of the factors that affect VE status, albeit stable over the lifespan, since other factors, such as VE dietary intake, dietary habits (e.g., consumption of other micronutrients) [61], oxidative stress (through e.g., smoking), and age [62] also affect this status. Thus, a prediction of VE status should take into account these variables as well.

## Figures and Tables

**Table 1:** Summary of SNPs associated with fasting blood VE concentration or VE bioavailability.

SNP	Global MAF ^1^	Nearest Gene	Trait	Reference	Study Type
rs12272004	0.085	*APOA5*	FBα	[52]	GWAS
rs964184	0.222	*APOA5*	FBα(αS)	[50]	GWAS
rs2108622	0.237	*CYP4F2*	FBα(αS)	[50]	GWAS
rs11057830	0.139	*SCARB1*	FBα(αS)	[50]	GWAS
rs7834588	0.433	*NKAIN3*	FBα(αS)	[50]	GWAS
rs10401969	0.118	*SUGP1*	FBα	[58]	GWAS
rs58542926	0.067	*TM6SF2*	FBα	[58]	GWAS
Rs675	0.099	*APOA4*	FBαγ	[22]	CGAS
E2, E3, E4	–	*APOE*	FBα	[22]	CGAS
rs4238001	0.064	*SCARB1*	FBγ	[22]	CGAS
rs5888	0.323	*SCARB1*	FBα	[22]	CGAS
rs662799	0.163	*APOA5*	FBα	[56]	CGAS
rs5128	0.234	*APOC3*	FBα	[21]	CGAS
rs708272	0.378	*CETP*	FBα	[21]	CGAS
rs1800588	0.387	*LIPC*	FBγ	[21]	CGAS
rs1527479	0.349	*CD36*	FBα	[57]	CGAS
rs6994076	0.349	*TTPA*	FBα	[54]	CGAS
rs2108622	0.237	*CYP4F2*	FBα(αS)	[55]	CGAS
rs3093105	0.157	*CYP4F2*	FBα(αS)	[55]	CGAS
rs468320	0.234	*ABCG1*	α-B ^2^	[23]	CGAS
rs2915775	0.257	*PNLIP*	α-B	[23]	CGAS
rs3010494	0.294	*PNLIP*	α-B	[23]	CGAS
rs1571513	0.240	*SLC10A2*	α-B	[23]	CGAS
rs9558203	0.198	*SLC10A2*	α-B	[23]	CGAS
rs16961116	0.162	*SLC10A2*	α-B	[23]	CGAS
rs12874168	0.210	*SLC10A2*	α-B	[23]	CGAS
rs2065550	0.160	*SLC10A2*	α-B	[23]	CGAS
rs2839715	0.168	*SREBF2*	α-B	[23]	CGAS
rs4822062	0.153	*SREBF2*	α-B	[23]	CGAS
rs4149314 *	0.069	*ABCA1*	α-B	[23]	CGAS
rs11789603 *	0.117	*ABCA1*	α-B	[23]	CGAS
rs2274873 *	0.082	*ABCA1*	α-B	[23]	CGAS
rs4149297 *	0.084	*ABCA1*	α-B	[23]	CGAS
rs4643493 *	0.082	*APOB*	α-B	[23]	CGAS
rs1042031 *	0.128	*APOB*	α-B	[23]	CGAS
rs1713222 *	0.155	*APOB*	α-B	[23]	CGAS
rs10464587 *	0.297	*BET1*	α-B	[23]	CGAS
rs1316328 *	0.134	*IRS1*	α-B	[23]	CGAS
rs4238329 *	0.148	*LIPC*	α-B	[23]	CGAS
rs8041525 *	0.086	*LIPC*	α-B	[23]	CGAS
rs7164909 *	0.153	*LIPC*	α-B	[23]	CGAS
rs8035357 *	0.150	*LIPC*	α-B	[23]	CGAS
rs12591216 *	0.084	*LIPC*	α-B	[23]	CGAS
rs12593880 *	0.068	*LIPC*	α-B	[23]	CGAS
rs4921920 *	0.101	*NAT2*	α-B	[23]	CGAS
rs7296124 *	0.107	*ZNF664*	α-B	[23]	CGAS
rs1048497 *	0.061	*ZNF664*	α-B	[23]	CGAS

^1^ Abbreviations: CGAS: candidate gene association study; GWAS: genome wide association study; MAF: minor allele frequency; The gene official symbols are those found in PubMed (available online: https://www.ncbi.nlm.nih.gov/gene/) and approved by the Hugo Gene Nomenclature Committee (available online: http://www.genenames.org/). FBα or FBγ: fasting blood α-tocopherol or γ-tocopherol concentration; FBα(αS): fasting blood α-tocopherol concentration following α-tocopherol supplementation; α-B: α-tocopherol bioavailability; ^2^ In this study, VE bioavailability was estimated by measuring the postprandial chylomicron α-tocopherol response (0 to 8 h area under the curve) to a α-tocopherol rich test-meal; * These SNPs were associated with the variability of α-tocopherol bioavailability, but this association was likely due to their involvement in the postprandial metabolism of chylomicrons [59], which are the lipoparticles that carry newly absorbed VE from the intestine to the liver.
